# QnrS1- and Aac(6′)-Ib-cr-Producing *Escherichia coli* among Isolates from Animals of Different Sources: Susceptibility and Genomic Characterization

**DOI:** 10.3389/fmicb.2016.00671

**Published:** 2016-05-23

**Authors:** Daniela Jones-Dias, Vera Manageiro, Rafael Graça, Daniel A. Sampaio, Teresa Albuquerque, Patrícia Themudo, Luís Vieira, Eugénia Ferreira, Lurdes Clemente, Manuela Caniça

**Affiliations:** ^1^National Reference Laboratory of Antibiotic Resistances and Healthcare Associated Infections, Department of Infectious Diseases, National Institute of Health Dr. Ricardo JorgeLisbon, Portugal; ^2^Centre for the Studies of Animal Science, Institute of Agrarian and Agri-Food Sciences and Technologies, Oporto UniversityOporto, Portugal; ^3^Innovation and Technology Unit, Human Genetics Department, National Institute of Health Dr. Ricardo JorgeLisbon, Portugal; ^4^Instituto Nacional de Investigação Agrária e VeterináriaLisboa, Portugal

**Keywords:** pathogenicity, *E. coli*, clone, PMQR, multidrug resistance, veterinary

## Abstract

*Salmonella enterica* and *Escherichia coli* can inhabit humans and animals from multiple origins. These bacteria are often associated with gastroenteritis in animals, being a frequent cause of resistant zoonotic infections. In fact, bacteria from animals can be transmitted to humans through the food chain and direct contact. In this study, we aimed to assess the antibiotic susceptibility of a collection of *S. enterica* and *E. coli* recovered from animals of different sources, performing a genomic comparison of the plasmid-mediated quinolone resistance (PMQR)-producing isolates detected. Antibiotic susceptibility testing revealed a high number of non-wild-type isolates for fluoroquinolones among *S. enterica* recovered from poultry isolates. In turn, the frequency of non-wild-type *E. coli* to nalidixic acid and ciprofloxacin was higher in food-producing animals than in companion or zoo animals. Globally, we detected two *qnrS1* and two *aac(6*′*)-Ib-cr* in *E. coli* isolates recovered from animals of different origins. The genomic characterization of QnrS1-producing *E. coli* showed high genomic similarity (O86:H12 and ST2297), although they have been recovered from a healthy turtle dove from a Zoo Park, and from a dog showing symptoms of infection. The *qnrS1* gene was encoded in a IncN plasmid, also carrying *bla*_TEM−1−_containing Tn*3*. Isolates harboring *aac(6*′*)-Ib-cr* were detected in two captive bottlenose dolphins, within a time span of two years. The additional antibiotic resistance genes of the two *aac(6*′*)-Ib-cr-*positive isolates (*bla*_OXA−1_, *bla*_TEM−1_,*bla*_CTX−M−15_, *catB3, aac(3)-IIa*, and *tetA*) were enclosed in IncFIA plasmids that differed in a single transposase and 60 single nucleotide variants. The isolates could be assigned to the same genetic sublineage—ST131 *fimH*30-Rx (O25:H4), confirming clonal spread. PMQR-producing isolates were associated with symptomatic and asymptomatic hosts, which highlight the aptitude of *E. coli* to act as silent vehicles, allowing the accumulation of antibiotic resistance genes, mobile genetic elements and other relevant pathogenicity determinants. Continuous monitoring of health and sick animals toward the presence of PMQR should be strongly encouraged in order to restrain the clonal spread of these antibiotic resistant strains.

## Introduction

Antibiotic resistance has been critically increasing over time and now constitutes one of the major health concerns worldwide. The uncontrolled use of antibiotics in human and veterinary practices, animal production and agriculture and the increasingly easiness in global transportation contributed to the dissemination of multidrug resistant pathogens that constitute a risk for humans, animals and the environment (Marshall and Levy, 2011; EFSA, 2015). Nowadays, antibiotic resistant *Salmonella enterica* and *Escherichia coli* are among the most problematic zoonotic bacteria, causing severe gastroenteritis in animals and humans (EFSA, 2015).

Fluoroquinolones constitute a group of broad spectrum antibiotics of critical importance, presenting applications in both human and veterinary medicines (Poirel et al., 2012). Therefore, resistance might easily emerge in animals and get transferred to humans through the food chain and direct or indirect contact. Several examples of such transmission have already been documented (Gomes-Neves et al., 2014; Damborg et al., 2015; Schmithausen et al., 2015). Fluoroquinolone resistance has emerged rapidly due to two main types of mechanisms: mutation of the chromosomal quinolone targets DNA gyrase and topoisomerase IV, and acquisition of the transferable plasmid-mediated quinolone resistance (PMQR) determinants *qnr, qepA, aac(6*′*)-Ib-cr*, and *oqxAB* (Veldman et al., 2011; Poirel et al., 2012). The alteration of chromosomal quinolone targets can lead to higher levels of resistance than PMQRs that are only able to guarantee low-level quinolone resistance. However, the ability of the latter to be spread by horizontal gene transfer constitutes a serious concern that should be addressed (Poirel et al., 2012). In fact, antibiotic resistance genes are frequently associated to mobile genetic elements such as insertion sequences (ISs), phages, transposons and plasmids, which enhance their ability to efficiently spread among different bacterial species (Stokes and Gillings, 2011). The most worrying mechanisms of resistance, which also show a transboundary spread between animals, humans and the environment, are, in fact, encoded by mobile antibiotic resistance genes. The occurrence of mobile genetic elements harboring multiple antibiotic resistance genes is also frequent, and enables the development of bacterial multidrug resistance, which may be responsible for therapeutic failures in animals or humans (Poirel et al., 2012).

In animals, as well as in humans, several factors can affect the progression and severity of an acute infection. The synchronized presence of antibiotic resistance genes, virulence factors, mobile genetic elements and other pathogenicity determinants, is ideal to the successful spread of these microorganisms in any environment (Cosentino et al., 2013).

In this study, PMQR-producing *E. coli* isolates were gathered from a collection of *S. enterica* and *E. coli* recovered from food-producing, companion and zoo animals, in the scope of their phenotypic and genotypic characterization. To further explore the genetic diversity of these isolates, as well as to understand the molecular features contributing to their spread and ability to cause infection, complete genomic sequencing was performed.

## Materials and methods

### Collection of bacterial isolates

This study included 89 *S. enterica* isolates recovered from breeders (*n* = 12), broilers (*n* = 33), layers (*n* = 33), swine (*n* = 6), and food products of animal origin (*n* = 5) (Table 1). In poultry farms, samples were collected from feces and environment using sterile boots/sock swabs. Food products included uncooked fresh products such as minced meat, hamburgers, meat cuts, sausages, and table eggs, randomly recovered at a variety of retail stores. Samples from other animal species (pigeons, partridges, ducks, pets, and exotic animals) consisted of blood cultures and organs (lung, liver, spleen, kidneys, and intestine) collected during post-mortem examination. All samples were examined according to ISO norm 6579:2002 applied to *Salmonella* detection in food and animal feeding stuffs. After biochemical confirmation, *Salmonella* spp. isolates were sent to the *Salmonella* National Reference Laboratory (INIAV, Lisbon) in triple sugar iron slopes or SMID plates.

**Table 1:** **Distribution of the *S. enterica*(*n* = 89) and *E. coli* (*n* = 91) isolates**.

**Source**	***S. enterica***			**Source**	***E. coli***
	**Enteritidis**	**Other serotypes^a^**	**Total**		
Breeders	12	0	12	Food	32
Layers	24	9	33	Companion	36
Broilers	32	1	33	Zoo	23
Swine	0	6	6		
Food of animal origin	3	2	5		
Total	71	18	89	Total	91

a *Salmonella 4,5:i:- (n = 1), Salmonella 6,7,14:-:1,2 (n = 1), Salmonella Bradenburg (n = 1), Salmonella Gallinarum (n = 1), Salmonella Give (n = 1), Salmonella Hadar (n = 1), Salmonella Heidelberg (n = 1), Salmonella IIIa 48:z10:- (n = 1), Salmonella Mbandaka (n = 1), Salmonella Rissen (n = 2), Salmonella Typhimurium (n = 3), Salmonella Virchow (n = 4)*.

This study also included 91 *E. coli* isolates (Table 1) collected from food-producing animals [(bovine, swine and poultry), (*n* = 32)], pets [(dogs, cats, horses, and cage birds), (*n* = 37)] and zoo animals [(terrestrial and aquatic mammals, birds and reptiles), (*n* = 22)]. Samples consisted of swabs from organic fluids and cavities, fecal samples, urine samples, blood cultures, and organs collected during post-mortem examination and submitted for bacteriological analysis. Suspected *E. coli* colonies obtained in MacConkey agar plates were confirmed by API 20E strips (bioMérieux, Marcy-l'Étoile, France).

### Serotypes of *S. enterica*

*S. enterica* isolates were serotyped by the slide agglutination method, using the method of Kauffmann-White scheme (Grimont and Weill, 2007).

### Antibiotic susceptibility testing

Minimum inhibitory concentrations (MICs) were determined by agar dilution following standard recommendations, using a panel of 10 antimicrobial compounds: ampicillin, cefotaxime, nalidixic acid, ciprofloxacin, gentamicin, streptomycin, chloramphenicol, tetracycline, sulfamethoxazole, and trimethoprim (Table 2). Isolates harboring PMQR determinants were further studied by determination of the MICs to a larger panel of fluoroquinolones, which included moxifloxacin, gatifloxacin, levofloxacin, ofloxacin, enrofloxacin, and norfloxacin. To assess non-wild-type isolates, interpretation of results was performed according to the epidemiological cut-off values suggested by the European Committee on Antimicrobial Susceptibility Testing (EUCAST, http://mic.eucast.org/Eucast2/). For *Salmonella* spp., the cut-off value used for sulfamethoxazole was that for sulfonamides from Clinical Standards Laboratory Institute (http://clsi.org). MIC_50_ and MIC_90_ were calculated as reported elsewhere (Schwarz et al., 2010). *E. coli* ATCC 25922 was used as the quality control strain. Isolates were considered multidrug resistant (MDR) if they presented non-wild-type phenotypes against three or more structurally unrelated antibiotics (Magiorakos et al., 2011).

**Table 2:** **MIC_50_ and MIC_90_ for *S. enterica* (*n* = 89) and *E. coli* (*n* = 91) isolates**.

**Antibiotic**	***S. enterica***				***E. coli***		
	**Food animals**				**Food animals (*n* = 32)**	**Zoo Animals (*n* = 23)**	**Companion Animals (*n* = 36)**
	**Breeders (*n* = 12)**	**Broilers (*n* = 33)**	**Layers (*n* = 33)**	**Others^a^(*n* = 11)**			
**Na**							
MIC_50_	128	128	128	128	8	4	4
MIC_90_	128	128	128	128	128	128	128
% Wt	0	12	6	18	59	77	78
%N-Wt	100	88	94	82	41	23	22
**Cp**							
MIC_50_	0.25	0.25	0.25	0.25	0.03	0.015	0.015
MIC_90_	0.25	0.25	0.25	0.5	8	8	8
% Wt	0	3	18	36	59	77	72
%N-Wt	100	97	82	64	41	23	28
**A**							
MIC_50_	2	4	0.5	8	8	8	8
MIC_90_	4	4	8	64	64	64	64
% Wt	100	100	94	64	53	50	53
%N-Wt	0	0	6	36	47	50	47
**Ct**							
MIC_50_	0.125	0.06	0.125	0.125	≤ 0.06	0.06	≤ 0.06
MIC_90_	0.125	0.125	0.125	2	0.125	0.125	0.125
% Wt	100	100	94	82	100	95	94
%N-Wt	0	0	6	18	0	5	6
**G**							
MIC_50_	0.25	0.25	0.25	0.5	0.5	0.5	0.5
MIC_90_	0.5	0.5	0.5	1	2	1	1
% Wt	100	100	100	100	91	91	100
%N-Wt	0	0	0	0	9	9	0
**St**							
MIC_50_	2	2	4	32	4	4	4
MIC_90_	8	4	128	64	256	256	128
% Wt	100	100	82	36	69	64	86
%N-Wt	0	0	18	64	31	36	14
**T**							
MIC_50_	2	2	2	4	32	2	2
MIC_90_	4	4	4	64	64	64	64
% Wt	92	100	91	55	47	59	75
%N-Wt	8	0	9	45	53	41	25
**C**							
MIC_50_	8	4	8	8	4	8	8
MIC_90_	8	8	8	16	64	8	16
% Wt	100	97	100	91	84	100	92
%N-Wt	0	3	0	9	16	0	8
**Su**							
MIC_50_	128	128	128	128	16	32	16
MIC_90_	128	128	128	>512	>512	>512	>512
% Wt	92	100	100	64	63	59	78
%N-Wt	8	0	0	36	38	41	22
**Tp**							
MIC_50_	0.5	0.5	0.5	0.25	0.5	0.5	0.5
MIC_90_	0.5	0.5	0.5	32	32	32	32
% Wt	92	100	100	73	81	68	81
%N-Wt	8	0	0	27	19	32	19

a *Others, pigs (n = 6) and food products of animal origin (n = 5)*.

*Na, nalidixic acid; Cp, ciprofloxacin; A, ampicillin; Ct, cefotaxime; G, gentamicin; St, streptomycin; T, tetracycline; C, chloramphenicol; Su, sulfamethoxazole; Tp, trimethoprim*.

### Molecular characterization of resistance

All isolates were evaluated regarding the presence of *qnrA, qnrB, qnrC, qnrD, qnrS, aac(6*′*)-Ib-cr*, and *qepA* genes, using primers and conditions previously described (Jones-Dias et al., 2013), and *oqxAB* genes using primers and conditions first described in this study (oqxA-F, 5′-AGAGTTCAAAGCCACGCTG-3′ and oqxb-R, 5′-CTCCTGCATCGCCGTCACCA-3′; initial denaturation of 94°C for 5 min; 94°C for 30 s, 64°C for 30 s and 72°C for 1 min, for 30 cycles; final step of extension of 72°C for 5 min). PMQR-producing isolates were also characterized regarding the production of β-lactamase-encoding genes and conventional Multilocus sequence typing (MLST), as described elsewhere (Jones-Dias et al., 2015).

### Genomic characterization of PMQR-producing *E. coli*

The genomes of the four PMQR-producing *E. coli* (LV46221, LV46743, LV36464, and LV27950) were characterized. Genomic DNA was extracted using DNeasy Blood and Tissue Kit (Qiagen, Aarhus) and quantified using Qubit 1.0 Fluorometer (Invitrogen, Waltham). The Nextera XT DNA Sample Preparation Kit (Illumina, San Diego, CA) was used to prepare sequencing libraries from 1 ng of genomic DNA, according to the manufacturer's instructions. Paired-end sequencing of 150 bp reads was performed on a MiSeq (Illumina). Sequence reads were then trimmed and filtered according to quality criteria, and assembled *de novo* using CLC genomics workbench version 8.5.1 (QIAGEN, Aarhus). RAST (Rapid Annotation using Subsystem Technology) was used for subsystem annotation of the genomes (Aziz et al., 2012; Overbeek et al., 2014).

### Identification of pathogenicity-related genes

Pathogenicity-related genes were detected using a variety of online web tools. PathogenFinder 1.1, ResFinder 2.1, VirulenceFinder 1.4, SerotypeFinder 1.1, MLST 1.8, pMLST 1.4, and PHAST were used to estimate the pathogenicity determinants, acquired antibiotic resistance genes, virulence factors, serotypes, MLST, plasmid MLST and phage regions, respectively in the genomes of PMQR-producing *E. coli* (Zhou et al., 2011; Larsen et al., 2012; Zankari et al., 2012; Cosentino et al., 2013; Carattoli et al., 2014; Joensen et al., 2014, 2015). ISsaga was also used to detect and annotate insertion sequences in the draft genomes of the *E. coli* isolates (Varani et al., 2011). Specific analysis of antibiotic resistance genes and respective flanking regions was carried out with CLC genomics workbench version 8.5.1 (Qiagen, Aarhus). Contigs carrying antibiotic resistance genes were manually assembled whenever necessary and blasted against GenBank to identify their genetic location.

### Nucleotide sequence genbank accession numbers

The draft genomes of isolates LV46221, LV46743, LV36464, and LV27950 have been deposited at DDBJ/EMBL/GenBank under the accessions LRXG00000000, LRXH00000000, LRXI00000000, and LRXJ00000000, respectively. The versions described in this paper are version LRXG01000000, LRXH01000000, LRXI01000000, and LRXJ01000000, respectively.

## Results

### Serotypes of *Salmonella* spp.

*S. enterica* serotype Enteritidis is one of the most common serotype in humans (EFSA, 2015) and it was the most frequently detected among the 89 *S. enterica* isolates (71/89, 79.8%), being present in all food animals except swine. The remaining *Salmonella* serotypes were detected in a less extent and were comprised of *Salmonella* 4,5:i:- (*n* = 1), *Salmonella* 6,7,14:-:1,2 (*n* = 1), *Salmonella* Bradenburg (*n* = 1), *Salmonella* Gallinarum (*n* = 1), *Salmonella* Give (*n* = 1), *Salmonella* Hadar (*n* = 1), *Salmonella* Heidelberg (*n* = 1), *Salmonella* IIIa 48:z10:- (*n* = 1), *Salmonella* Mbandaka (*n* = 1), *Salmonella* Rissen (*n* = 2), *Salmonella* Typhimurium (*n* = 3), and *Salmonella* Virchow (*n* = 4).

### Antimicrobial susceptibility of *S. enterica* and *E. coli* isolates

Susceptibility profiles of *S. enterica* and *E. coli* isolates differed with the animal group (Table 2). Although, high rates of non-wild-type *S. enterica* were detected for nalidixic acid (from 82 to 100%) and ciprofloxacin (from 64 to 100%) in all groups, they were particularly evident in poultry, and predominant in breeders. *S. enterica* isolates recovered from other sources (swine and food products, *n* = 11), showed higher non-wild-type phenotypes for ampicillin (36%), streptomycin (64%), tetracycline (45%), sulfamethoxazole (36%), and trimethoprim (27%) (Table 2). The poultry groups of breeders and broilers were mainly susceptible to ampicillin (100%), cefotaxime (100%), gentamicin (100%), and streptomycin (100%).

The frequency of non-wild-type isolates was globally higher for *E. coli* than for *S. enterica* against ampicillin (minimum value of 47 vs. 0%, respectively), tetracycline (minimum value of 25 vs. 0%, respectively), sulfamethoxazole (minimum value of 22 vs. 0%, respectively) and trimethoprim (minimum value of 19 vs. 0%, respectively). Although, no major discrepancies were noticed for *E. coli* in rates of non-wild-type isolates for the different animal groups, isolates recovered from food animals still presented more non-wild-type phenotypes than zoo or companion animals against nalidixic acid (41%), ciprofloxacin (41%), tetracycline (53%), and chloramphenicol (16%).

In *S. enterica* isolates from poultry, similar MIC_50_ and MIC_90_ values were observed for the majority of the antibiotics tested; major differences (≥3 fold dilutions) were observed for the group “others” for ampicillin (8 and 64 mg/L), tetracycline (4 and 64 mg/L), sulfamethoxazole (128 and >512 mg/L) and trimethoprim (0.25 and 32 mg/L). For *E. coli*, the most significant differences in MIC_50_ and MIC_90_ values were observed for nalidixic acid (4 and 128 mg/L), ampicillin (8 and 64 mg/L), streptomycin (4 and 128 mg/L), tetracycline (2 and 64 mg/L), sulfamethoxazole (16 and >512 mg/L) and trimethoprim (0.5–32 mg/L).

While for *S. enterica* only 9.0% (8/89) MDR isolates were detected, for *E. coli*, MDR was registered in 38.9% (35/90) of the isolates, which were distributed among 16/90 isolates from food-producing animals, 9/90 isolates from companion animals, and 10/90 isolates from zoo animals.

### Molecular characterization of *S. enterica* and *E. coli* isolates

Overall, among the 180 isolates studied, we have detected and identified four PMQR determinants in *E. coli* isolates: two *qnrS1* were detected in isolates recovered from a captive turtle dove (LV46221) and a pet dog (LV46743), and two *aac(6*′*)-Ib-cr* were isolated from *E. coli* recovered from captive bottlenose dolphins (LV36464 and LV27950). The detection of β-lactamase-encoding genes showed the presence of *bla*_TEM−1_in isolates LV46221 and LV46743, and *bla*_TEM−1_, *bla*_OXA−1_, and *bla*_CTX−M−15_ in LV36464 and LV27950. No other PMQR- or β-lactamase-encoding genes were identified in the collection of *E. coli* and *S. enterica* isolates.

### Genomic characterization of qnrS1-producing *E. coli*

The assembly of the genome sequences of the two *qnrS1*-harboring *E. coli*, LV46221 and LV46743, yielded 200 and 199 contigs (each >200 bp long), which together comprised 4,799,985 bp and 4,801,518 bp, respectively. The average coverage of LV46221 was 135.9, while LV46743 displayed 114.1 fold. The maximum contig length obtained for these genomes was 398,205 bp and 333,601 bp, respectively (Table 3).

**Table 3:** **Genome analysis of *E. coli* LV46221, LV46743, LV36464, and LV27950**.

**Isolates**	**LV46221**	**LV46743**	**LV36464**	**LV27950**
Genome size (bp)	4,799,985	4,801,518	5,180,399	5,156,819
Number of contigs	200	199	136	209
Average coverage	135.9	114.1	178.7	150.1
N50 (bp)^a^	119,356	119,356	158,975	158,977
Maximum contig (bp)	398,205	333,601	399,998	369,918
Minimum contig (bp)	208	201	486	218
Protein-coding genes	4618	4609	5107	5069
RNAs	77	86	77	76

a *Minimum contig length of at least 50% of the contigs*.

The automated annotation of the draft genomes showed that LV46221 (63%, 2879/4618) and LV46743 (63%, 2873/4609) presented a similar number of sequences attributed to specific subsystems (Figure S1). General annotation of both genomes showed 109 coding sequences associated with virulence, disease and defense, as well as 143 sequences coding for functions related with mobile genetic elements, such as phages, prophages, transposable elements, and plasmids. Globally, the proportion of each subsystem was equally represented in the genomes of the two isolates (Figure S1). According to RAST annotation system, LV46221 and LV46743 isolates carried 77 and 86 RNAs, respectively. The bioinformatics analysis of the genetic relatedness was carried out with regard to serotype and MLST: the serotypes of both isolates were defined as O86:H12, and they also shared the assigned MLST–ST2297 (Table 4).

**Table 4:** **General features of PMQR-harboring *E. coli* isolates recovered from animals of different sources**.

**Isolate**	**Origin**	**Year**	**Serotype**	**MIC (mg/L)**								**PMQR**	**Other resistance genes**	**Virulence factors**	**MLST**	**Plasmids**	**pMLST**
				**Mx**	**Cp**	**Ga**	**Le**	**Of**	**Ef**	**Na**	**Nx**						
LV46221	Dove	2008	O86:H12	0.75	0.38	0.75	0.38	1	1.5	8	0.75	*qnrS1*	*bla*_TEM−1_	*gad, iss*	ST2297	IncN	ST1
LV46743	Dog	2008	O86:H12	0.5	0.38	0.5	0.5	1.5	2	8	1.5	*qnrS1*	*bla*_TEM−1_	*gad*	ST2297	IncN	ST1
LV36464	Dolphin	2009	O25:H4	>32	>32	8	>32	>32	>32	>256	>256	*aac(6′)-Ib-cr*	*aac(3)-IIa, bla*_CTX−M−15_ *bla*_TEM−1_,*bla*_OXA−1_ *catB3, tetA*	*iss, sat, gad*	ST131	IncFIA, IncX	F-:A1:B-
LV27950	Dolphin	2011	O25:H4	12	>32	8	8	>32	>32	>256	>256	*aac(6′)-Ib-cr*	*aac(3)-IIa, bla*_CTX−M−15_ *bla*_TEM−1_,*bla*_OXA−1_ *catB3, tetA*	*iss, sat*	ST131	IncFIA	F-:A1:B-

*Mx, moxifloxacin; Cp, ciprofloxacin; Ga, gatifloxacin; Le, levofloxacin; Of, ofloxacin; Ef, enrofloxacin; Na, nalidixic acid; Nx, norfloxacin*.

*PMQR, Plasmid-mediated quinolone resistance*.

*MLST, Multilocus sequence typing*.

*In silico* analysis of the antibiotic resistance genes (90% identity and 40% minimum length) confirmed the presence of a *qnrS1*, and identified *bla*_TEM−1_gene in both isolates (Table 4). *qnrS1* was detected in a contig with an approximate length of 11,000 bp in both cases, showing 99% of homology with a resistance region from *S. enterica* subsp. *enterica* serovar Infantis pINF5 plasmid. By mapping all contigs against this plasmid, we detected *bla*_TEM−1_-containing T*n3*, and a disrupted IS*2*-like element upstream of *qnrS1*, as well as IS*26* transposase downstream of the gene. Other contigs showed complementary regions, revealing the presence of a fragment encoding conjugation transfer genes upstream of Tn*3* that showed homology with *Salmonella* Virchow plasmid pVQS1 (99%).

LV46221 and LV46743 showed no additional PMQR or other acquired antibiotic resistance genes. Moreover, no mutations were detected in the quinolone resistance determining region (QRDR) of genes *gyrA, gyrB, parC*, and *parE*, which are known to confer high level resistance to fluoroquinolones (Veldman et al., 2011). The isolates were also characterized with regard to specific mobile genetic elements of different classes. The screening of typable plasmids (>98% homology) enabled the identification of IncN plasmids, which were further typed as ST1 by pMLST (Table 4). ISsaga allowed the specialized annotation of insertion sequences and revealed a different distribution of the same elements for LV46221 and LV46743: IS*1* (3.23 and 1.96%, respectively), IS*200*_IS*605* (3.23 and 3.92%, respectively), IS*21* (3.23 and 3.92%, respectively), IS*3* (24.19 and 21.57%, respectively), IS*4* (3.23 and 3.92%, respectively), IS*481* (1.61 and 1.96%, respectively), IS*5* (1.61 and 1.96%, respectively), IS*6* (1.61 and 1.96%, respectively), IS*As1*(27.42 and 27.45%, respectively), IS*Kra4* (6.45 and 5.88%, respectively), IS*L3* (12.9 and 9.8%, respectively), ISNCY (9.68 and 11.76%, respectively), and finally Tn*3* (1.61 and 1.96%, respectively). IS*As1* was the most frequent element detected in both isolates, and IS*66* was exclusively detected in LV46743 (1.96%). Moreover, in LV46221 we identified 10 prophage regions among which three were questionable but seven were intact. The latter included prophage regions reaching up to 90.6Kb, containing 133 coding sequences (Table S1). In turn, in LV46743, 14 different prophages were detected that included one questionable, four incomplete and nine intact phage regions; intact zones ranged between regions of 10.4 Kb carrying 12 coding sequences, and 70.3 Kb with 88 protein coding DNA fragments. Overall, among the prophages showing higher scores for both genomes were serotype-converting *Shigella flexnerri* bacteriophage and Enterobacteria lambda phages (Table S1).

The total number of pathogenicity determinants, which according with PathogenFinder includes, for instance, virulence factors, antibiotic resistance genes and mobile genetic elements, detected a similar number of sequences in the genomes of *E. coli* LV46221 and LV46743: 607 and 611 different pathogenic families showed a 93.5% certainty of the isolates being human pathogens. Finally, the virulence factor glutamate decarboxylase (*gad*) was detected in both isolates, while the increased serum survival factor *iss* was exclusively identified in LV46221 (Table 4).

### Genomic characterization of Aac(6′)-ib-cr-Producing *E. coli*

The genome sequences of isolates LV36464 and LV27950, which were known to produce Aac(6′)-Ib-cr and CTX-M-15, were also compared. Their *de novo* assembly yielded 5,180,399 bp for LV36464 and 5,156,819 bp for LV27950 and displayed a mean coverage of 178.6 and 150.1 fold, respectively. Approximately, 136 and 209 contigs (each >200 bp long) were recovered for LV36464 and LV27950 with a maximum contig length of 399,998 and 369,918 bp, respectively (Table 3).

The automated annotation of the genomes showed a total number of coding sequences of 5107 for LV36464 and 5069 for LV27950, excluding 77 and 76 annotated RNA molecules. The distribution of the annotated coding sequences by subsystem showed an identical representation of functions in both isolates (LV36464: 368%, 3062/5107; LV27950: 61%, 3081/5069; Figure S2).

The serotypes of the LV36464 and LV27950 isolates obtained upon the analysis of *fliC, wzy*, and *wzx* genes, were defined as O25:H4. The epidemiology and diversity of *E. coli* isolates was also explored, assigning both of them to ST131 and to sublineage *fimH*30-Rx.

Globally, in isolates LV36464 and LV27950 seven different acquired antibiotic resistance genes were detected: *aac(6*′*)Ib-cr, bla*_OXA−1_, *bla*_TEM−1_,*bla*_CTX−M−15_, *catB3, aac(3)-IIa*, and *tetA*. By mapping, the main difference between the plasmids carried by these isolates was the deletion of a 2400 bp sequence that displayed 99.7% homology with the transposase of Tn*5403*. Moreover, 60 single nucleotide variants have been detected between them. Both plasmids displayed an IncF plasmid from a ST131 *E. coli* isolate (JJ2434, unpublished) as its best blast hit. The comparative analysis with JJ2434 showed the absence of two regions of 9329 and 1740 bp that corresponded to deletions of genes coding for unknown functions, replication proteins, endonucleases, transcriptional regulators, and conjugation transfer proteins in LV36464 and LV27950 plasmids. The analysis of the QRDR of genes *gyrA* (from 67 to 106 aminoacids), *gyrB* (from 415 to 470 aminoacids), *parC* (from 47 to 133 aminoacids), and *parE* (from 450 to 528 aminoacids) revealed the presence of amino acid substitutions in *gyrA* (S83L and D87N) and *parC* (S80I and E84V) in both isolates.

A high number of mobile genetic elements was detected in the draft genomes of these isolates. Both harbored a plasmid (>98% homology) from incompatibility group IncFIA, which according to PlasmidFinder was classified as an IncFIA type 1. LV36464 accommodated an additional IncX plasmid. The distribution of insertion sequences present in LV36464 and LV27950 genomes was also globally similar: IS*1* (5.77 and 5.66%, respectively), IS*110* (3.85 and 1.89%, respectively), IS*1380* (1.92 and 1.89%, respectively), IS*200*_IS*605* (5.77 and 3.77%, respectively), IS*21* (3.85 and 3.77%, respectively), IS*3* (23.08 and 26.42%, respectively), IS*30* (1.92 and 3.77%, respectively), IS*4* (5.77 and 3.77%, respectively), IS*481* (3.85 and 3.77%, respectively), IS*6* (1.92 and 1.89%, respectively), IS*66* (11.54 and 11.32%, respectively), IS*As1* (1.92 and 1.89%, respectively), IS*L3* (19.23 and 18.87%, respectively) and IS*NCY* (9.62 and 9.43%, respectively). It is worth mentioning that the worldwide disseminated Tn*3* was only represented in the genome of LV36464 (3.39%), and IS*92* (1.89%) in LV27950. The specialized annotation of phage and prophages revealed that LV36464 harbored 17 regions: 8 intact, 6 incomplete and 4 questionable. These intact prophage regions ranged between 17.4 and 51.5 Kb, showing different numbers of coding sequences that varied between 24 and 88. In turn, LV27950 harbored 13 prophage regions: it displayed 10 intact regions spanning between 20.6 and 86.1 Kb. Globally, regions from five phages were present in the genomes of both and two were exclusive of each isolate (Table S1).

The detection of virulence factors in the genome of LV27950 revealed the presence of an increased serum survival factor provided by an ISS-encoding gene and a secreted autotransporter toxin denominated *sat* (Table 4). LV36464 shared the same virulence factors and, in addition, harbored a glutamate descarboxilase-encoding gene (*gad*). The overall estimation of pathogenicity factors present in the genome of the isolates, using known proteins with recognized involvement in pathogenicity as reference, enabled us to determine that the assembled contigs of LV36464 and LV27950 matched 553 and 544 pathogenic families, which resulted in the estimation of both isolates being human pathogens (93.1 and 93.3%), confirming their zoonotic potential.

## Discussion

The prevalence of antibiotic resistance genes in isolates from animal origin has been fairly assessed (Szmolka et al., 2011; Tamang et al., 2011; Bardon et al., 2013; Clemente et al., 2015). However, taking in account the current availability of genomic characterization tools, we are now able to proceed with more detailed characterizations of these genes, in a broader context. In this study, we characterized the genome of PMQR-producing *E. coli*. To understand the antibiotic susceptibility background of these specific isolates we have also evaluated the antibiotic susceptibility phenotypes of a collection of *S. enterica* and *E. coli* recovered from animals of different origins, in which the isolates were originally included.

The levels of non-wild type phenotypes revealed to be very distinct among *S. enterica* and *E. coli*. Non wild-type isolates for fluoroquinolones were particularly evident among poultry isolates recovered from *S. enterica*. Regarding *E. coli* isolates, the frequency of non-wild-type phenotypes to nalidixic acid and ciprofloxacin was higher in food-producing animals than in companion and zoo animals, which might be due to the high consumption of veterinary antibiotics in animal industrial units, particularly tetracyclines, sulphonamides, and fluoroquinolones (EFSA, 2015). Portugal still represents a European country with high antibiotic use in animals. This fact raises concerns regarding antibiotic resistance in veterinary settings (EMA, 2014). Different MIC_50_ and MIC_90_ (3-fold dilutions) were noted for some groups of each species: *E. coli* isolates for nalidixic acid, ciprofloxacin, ampicillin, streptomycin, tetracycline, sulfamethoxazole and trimethoprim, and *S. enterica* for ampicillin, cefotaxime, tetracycline, and trimethoprim.

Although, PMQR determinants are typically responsible by low level resistance, their presence has been increasingly reported in animals, resulting in an additional effect on the nonsusceptibility of fluoroquinolones (Ahmed and Shimamoto, 2013; Donati et al., 2014; Jamborova et al., 2015). The high MIC values of 128 mg/L against nalidixic acid and 8 mg/L against ciprofloxacin observed in some of the isolates of our collection may be associated with amino acid alterations in the quinolone resistance-determining region (QRDR). Indeed, although the fluoroquinolone non-susceptibility is frequently compromised by target modification, the PMQR-encoding genes have the potential to spread and promote co-selection of other antibiotic resistance genes (EMA, 2014). Late reports even suggest that the spread of PMQR may not be triggered by selection pressure, which justifies the low rates of these determinants in animals, despite the high use of fluoroquinolones (Veldman et al., 2011).

Considering the high level MICs, most likely caused by QRDR chromosomal mutations that might mask the presence of PMQR, we decided in this study to retrospectively search for these determinants in all isolates of the collection, regardless of the MIC value. We have detected four PMQR-encoding genes (4/180) (two *qnrS1* and two *aac(6*′*)-Ib-cr*) in *E. coli* LV46221, LV46743, LV36464, and LV27950 recovered from animals of different origins: a healthy turtle dove from a Zoo Park (2008), a diseased pet dog (2008), a bottlenose dolphin from a Zoo Park showing signs of respiratory infection (2009), and a second but healthy bottlenose dolphin from the same Zoo Park (2011) (Table 4).

The comparison of the genomes of QnrS1-producing *E. coli* revealed that isolates LV46221 and LV46743 were very similar in terms of their global pathogenicity potential, although they were recovered from animals of different classes and completely different backgrounds (Table 4). The absence of chromosomal mutations in the QRDR of isolates LV46221 and LV46743 corroborated the low fluoroquinolone MIC values obtained, which spanned between 0.38 mg/L for ciprofloxacin and 8 mg/L for nalidixic acid, highlighting the low level resistance conferred by QnrS1 determinants (Cavaco and Aarestrup, 2009). The plasmid region in which the *qnrS1* was enclosed in both isolates, that included the association with Tn*3*, has already been described in association with *qnrS1* genes in plasmids from *Shigella flexneri* recovered from food products, *Salmonella* Infantis from avian origin, and human clinical *Klebsiella pneumoniae* isolates, respectively (Hata et al., 2005; Chen et al., 2006; Kehrenberg et al., 2006). Moreover, we have previously detected other *qnrS1* from animals in Portugal, associated with a similar genetic environment, exclusively in food-producing animals (Jones-Dias et al., 2013). IncN plasmids harbored by LV46221 and LV46743 were assigned to ST1 by pMLST, which have also been associated with chickens and wild bird water in Czech Republic and the Netherlands, respectively (Ben Sallem et al., 2014).

Few genomic differences were noticed between the two *aac(6*′*)-Ib-cr-* and *bla*_CTX−M−15_-harboring *E. coli*. In fact, the isolates could be assigned to the same genetic sublineage—ST131 *fimH*30-Rx, confirming clonal spread. Although, samples have been recovered within a reasonable time span of 2 years, their origin refers to two bottlenose dolphins of the same species held captive in the same Zoo Park. The presence of four chromosomal alterations in the QRDR region of isolates LV36464 and LV27950 was reflected in the high levels of fluoroquinolone MICs, which ranged between 8 and >256 mg/L. All antibiotic resistance genes detected in LV36464 and LV27950 [*aac(6*′*)Ib-cr, bla*_OXA−1_, *bla*_TEM−1_,*bla*_CTX−M−15_, *catB3, aac(3)-IIa*, and *tetA*] could be traced back to a single multidrug resistance IncFIA plasmid that showed 99.9% of homology with a plasmid submitted this year to Genbank in U.S.A (JJ2434, unpublished). Although, 60 single nucleotide variants have been detected between the LV36464 and LV27950 plasmids, the main difference consisted of a single deletion that involved part of a transposase-encoding gene. The absence of a set of conjugation transfer proteins (*tra* genes), among other genes, highlighted the preponderance of clonal spread over horizontal gene transfer in ST131 *E. coli* (Nicolas-Chanoine et al., 2014). Although, several isoforms of identical plasmids have been detected worldwide, the simultaneous resistance to β-lactams, fluoroquinolones, aminoglycosides, chloramphenicol and tetracyclines has been a permanent feature, which reinforces the advantage that it confers (Boyd et al., 2004; Zhou et al., 2015). The detection of a ST131 *fimH*30-Rx *E. coli* in two dolphins, which are continuously in contact with a live audience, constitutes a public health concern. These clinically relevant multidrug resistant *E. coli* isolates have been on the rise for years (Nicolas-Chanoine et al., 2014). Initially restricted to clinical contexts, recent findings suggest that their prevalence in non-clinical settings is maintained by the constant exchange of isolates throughout the time, as verified in this study (Mathers et al., 2015).

Although, *E. coli* is a common inhabitant of the gastrointestinal tract of humans and animals, the detected transposons, plasmids and bacteriophages are essential to the acquisition of pathogenicity factors that enlarge their ability to adapt to new niches, allowing bacteria to increase the capacity to cause a broad spectrum of diseases (Bien et al., 2012). All isolates displayed genomic factors that may be critical to cause a zoonotic infection and that were reflected in high probabilities for the isolates to be human pathogens (>93%). Concerning virulence factors, we detected the presence of glutamate decarboxylase, increased serum survival gene and a secreted autotransporter toxin, irregularly distributed across the four isolates (Table 3), which did not denote any relation with the conditions of their respective hosts. These virulence factors confer resistance to extreme acid conditions of the intestines, enable the isolate to survive complement system and cause defined damage to kidney epithelium, being indicative of their ability to cause disease (Johnson et al., 2008; Becker Saidenberg et al., 2012). Indeed, *E. coli* isolates can frequently encode a number of virulence factors, which enable the bacteria to colonize the urinary tract and face highly effective host defenses (Bien et al., 2012).

Although, fluoroquinolones are consistently used in veterinary medicine, results presented in this study indicate that PMQR determinants occurred at a low frequency in these isolates (2.2%), as previously reported (Donati et al., 2014; Jamborova et al., 2015). However, the studied groups of animals should still be considered potential reservoirs for PMQR-producing isolates, especially because there is the inherent potential for transboundary dissemination. These isolates presented a set of genetic features essential to promote their own successful spread: multiple antibiotic resistance genes carried by well-known mobile genetic elements, virulence factors adequate to zoonotic transmission and numerous other pathogenicity factors.

The analysis of many bacterial genomic features showed us great genetic relatedness between the two *qnrS1*- and *aac(6*′*)-Ib-cr*-harboring isolates. The data gathered throughout this study illustrates two scenarios: the presence of the same strain in different hosts inhabiting remote locations and the persistence of a unique strain in a single niche during a long period of time. The strains were each associated with a case of symptomatic infection (LV46743 and LV36464) and with a report of microbiological control of an asymptomatic host (LV46221 and LV27950), which reinforces the ability of *E. coli* isolates to act as silent vehicles, allowing the accumulation of antibiotic resistance determinants, mobile genetic elements and other relevant pathogenicity determinants (Mathers et al., 2015). It is not certain whether these bacteria spread from humans to animals, between different animals or from the environment to animals. However, in the case of companion animals, but particularly zoo animals, surveillance is essential to prevent continuous dissemination. The contact between animals and owners, zookeepers, visitors, and handlers raises concerns, considering that these bacteria might easily spread to humans and to other animals (Veldman et al., 2011; Ewers et al., 2012).

Permanent surveillance of health and sick animals should be strongly encouraged, regardless of their origin, in order to monitor future trends in the dissemination of resistance to fluoroquinolones and other antibiotics. Furthermore, this genome project contains valuable scientific data which will certainly be helpful for molecular epidemiological surveys of ST131 *E. coli* clonal group.

## Author contributions

DJD designed the study, acquired laboratory data, analyzed the data and wrote the manuscript. VM designed the study, analyzed the data and reviewed the manuscript. RG acquired laboratory data. DAS acquired laboratory data, TA, acquired laboratory or epidemiological data. PT acquired laboratory or epidemiological data. LV acquired laboratory data and reviewed the manuscript. EF acquired laboratory data and reviewed the manuscript. LC acquired laboratory or epidemiological data and reviewed the manuscript. MC designed the study and reviewed the manuscript. All authors read and approved the final manuscript.

## Funding

These work was supported by Fundação para a Ciência e a Tecnologia (grant numbers PTDC/CVT/65713 and PEst-OE/AGR/UI0211/2011-2014). DJD has received research funding from Funda73o para a Cie a Tecnologia (FCT, grant number SFRH/BD/80001/2011). VM was supported by FCT fellowship (grant SFRH/BPD/77486/2011), financed by the European Social Funds (COMPETE-FEDER) and national funds of the Portuguese Ministry of Education and Science (POPH-QREN).

### Conflict of interest statement

The authors declare that the research was conducted in the absence of any commercial or financial relationships that could be construed as a potential conflict of interest.
