# Supplementary material for: QnrS1- and Aac(6′)-Ib-cr-Producing Escherichia coli among Isolates from Animals of Different Sources: Susceptibility and Genomic Characterization
Source: Front Microbiol. 2016 May 23;7:671. doi: 10.3389/fmicb.2016.00671 (PMC4876607; doi:10.3389/fmicb.2016.00671)
Supplement: Supplementary file 1 [file Table1.docx]

**Table S1.** Representation of intact phage regions detected in the draft genome of LV46221, LV46743, LV36464 and LV27950.

| **Phage regions** | **Region length (Kb)** | **Score** | **Number of coding sequences** | **Accession number** |
| --- | --- | --- | --- | --- |
| **LV46221** | | | | |
| 1 | 30.4 | 100 | 36 | NC_003315 |
| 2 | 19.4 | 100 | 23 | NC_016158 |
| 3 | 14.7 | 100 | 17 | NC_001416 |
| 4 | 58.9 | 150 | 67 | NC_021857 |
| 5 | 54.1 | 150 | 50 | NC_010463 |
| 6 | 37.9 | 150 | 39 | NC_001416 |
| 7 | 90.6 | 150 | 133 | NC_001416 |
| **LV46743** | | | | |
| 1 | 10.4 | 96 | 12 | NC_001609 |
| 2 | 19 | 100 | 21 | NC_001416 |
| 3 | 24.7 | 100 | 35 | NC_010463 |
| 4 | 18.6 | 110 | 28 | NC_028943 |
| 5 | 54.5 | 100 | 37 | NC_022747 |
| 6 | 48.4 | 150 | 56 | NC_021857 |
| 7 | 21.9 | 130 | 29 | NC_016158 |
| 8 | 43 | 150 | 44 | NC_001416 |
| 9 | 70.3 | 150 | 88 | NC_001416 |
| **LV36464** | | | | |
| 1 | 33.9 | 150 | 46 | NC_001895 |
| 2 | 46.8 | 120 | 34 | NC_026014 |
| 3 | 36.8 | 93 | 54 | NC_009237 |
| 4 | 31.8 | 150 | 36 | NC_001416 |
| 5 | 49.4 | 150 | 61 | NC_019522 |
| 6 | 17.4 | 140 | 24 | NC_004813 |
| 7 | 44.7 | 150 | 38 | NC_004813 |
| 8 | 51.1 | 150 | 88 | NC_019716 |
| **LV27950** | | | | |
| 1 | 68.5 | 150 | 80 | NC_019522 |
| 2 | 34.7 | 150 | 26 | NC_019716 |
| 3 | 33.9 | 150 | 44 | NC_022750 |
| 4 | 40.6 | 120 | 32 | NC_026014 |
| 5 | 34.7 | 150 | 46 | NC_005882 |
| 6 | 25.3 | 150 | 29 | NC_004813 |
| 7 | 25.2 | 110 | 38 | NC_001416 |
| 8 | 20.6 | 150 | 30 | NC_001416 |
| 9 | 58.3 | 150 | 90 | NC_019716 |
| 10 | 86.1 | 150 | 169 | NC_001416 |
