# Supplementary material for: QnrS1- and Aac(6′)-Ib-cr-Producing Escherichia coli among Isolates from Animals of Different Sources: Susceptibility and Genomic Characterization
Source: Front Microbiol. 2016 May 23;7:671. doi: 10.3389/fmicb.2016.00671 (PMC4876607; doi:10.3389/fmicb.2016.00671)
Supplement: Supplementary file 2 [file Image1.PDF]

A

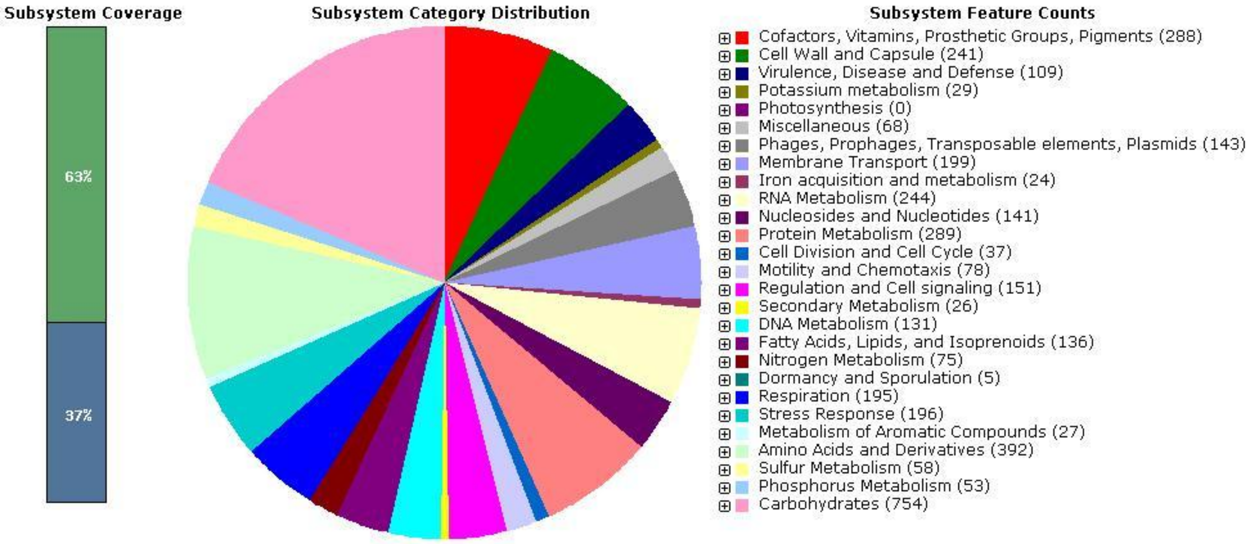

B

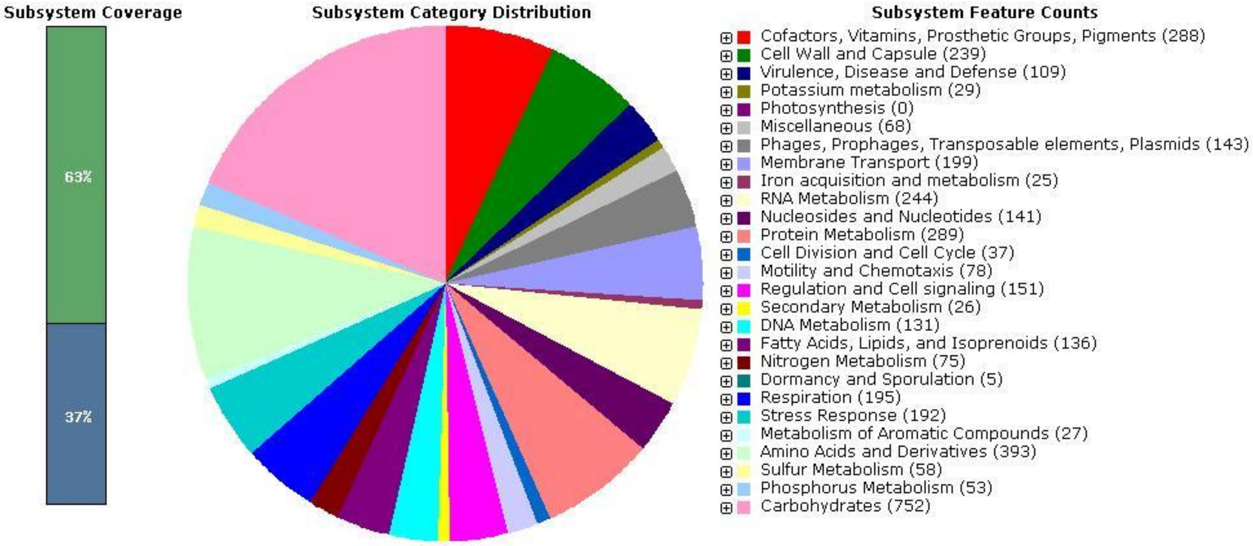

Figure S1. Representation of relative abundance of each subsystem category in isolates LV46221 (A) and LV46743 (B).

A

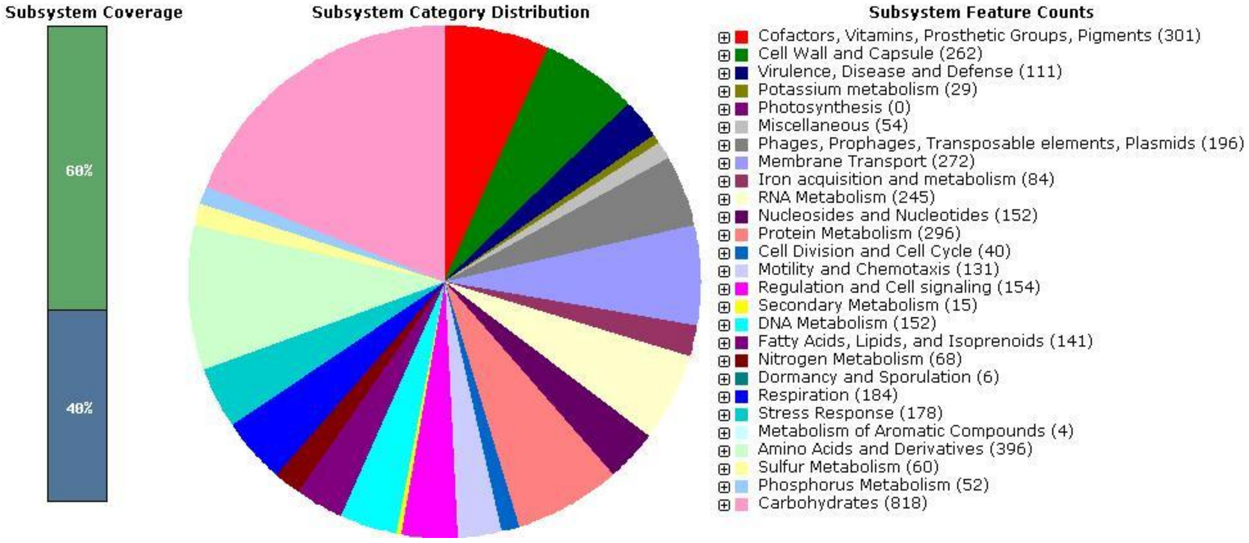

B

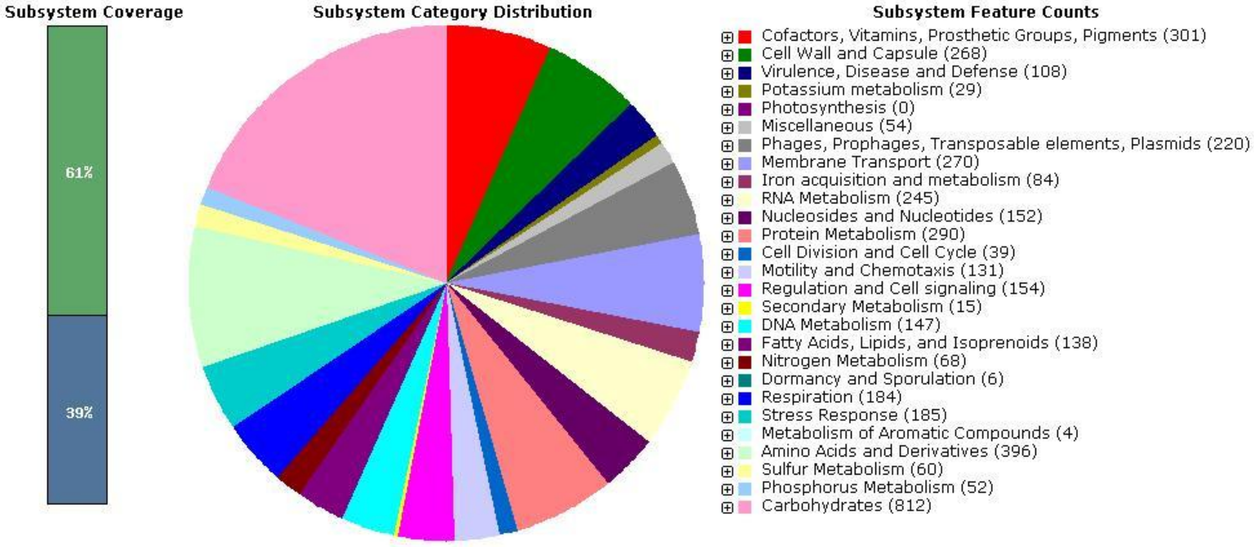

Figure S2. Representation of relative abundance of each subsystem category in isolates LV36464 (A) and LV27950 (B).
